# The role of the prognostic nutritional index in predicting mortality in stroke patients

**DOI:** 10.1590/1806-9282.20240714

**Published:** 2024-09-13

**Authors:** İzzet Ustaalioğlu, Gülbin Aydoğdu Umaç

**Affiliations:** 1 Gönen State Hospital, Department of Emergency Medicine – Balıkesir, Turkey.; 2 Manisa Provincial Ambulance Service Chief Physician – Manisa, Turkey.

**Keywords:** Prognostic nutritional index, Acute ischemic stroke, Mortality, Inflammation

## Abstract

**OBJECTIVE:**

The aim of this study was to evaluate the role of the prognostic nutritional index in predicting in-hospital mortality among patients with acute ischemic stroke.

**METHODS:**

This retrospective, observational study included patients diagnosed with acute ischemic stroke at the emergency department of the hospital between January 1, 2022, and January 1, 2023. Demographic data, vital parameters, comorbidities, stroke interventions, and laboratory data were collected from electronic medical records. Prognostic nutritional index was calculated using serum albumin levels and a total lymphocyte count. The primary outcome was in-hospital mortality.

**RESULTS:**

The study included 176 patients, divided into survivor (93.2%, n=164) and deceased (6.8%, n=12) groups. No significant differences were observed in age, gender, blood pressure, heart rate, or body temperature between the groups. Atrial fibrillation was significantly more common in the deceased group (50%) compared to the survivor group (18.9%) (p=0.011). The median lymphocyte count was significantly higher in the survivor group (1,353 [interquartile range, IQR 984–1,968]/mm³) compared to the deceased group (660 [IQR 462–1,188]/mm³) (p=0.009). The median albumin level was significantly lower in the deceased group (3.31 [IQR 2.67–3.4] g/dL) compared to the survivor group (3.74 [IQR 3.39–4.21] g/dL) (p<0.001). The median prognostic nutritional index was significantly higher in the survivor group (46.05 [IQR 39.1–51.3]) compared to the deceased group (36.7 [IQR 28.7–40.5]) (p<0.001). The area under the receiver operating characteristic for prognostic nutritional index predicting mortality was 0.791 (95%CI 0.723–0.848) (p=0.0002), with a cut-off value of ≤41.92 providing the highest diagnostic accuracy.

**CONCLUSIONS:**

Prognostic nutritional index is a valuable prognostic indicator for in-hospital mortality in acute ischemic stroke patients. Low prognostic nutritional index values are associated with increased mortality risk. Incorporating prognostic nutritional index into clinical practice may aid in the early identification of high-risk patients and the optimization of treatment strategies. Further research is needed to validate these findings and explore the broader clinical applications of prognostic nutritional index.

## INTRODUCTION

Stroke is one of the leading causes of death and permanent ­disability worldwide, posing a significant health burden on both patients and society^1-4^. Determining the prognosis of stroke patients is crucial for optimizing treatment strategies and ­effectively ­utilizing resources. In this context, prognostic models have been developed using various biochemical and clinical parameters.

Stroke is characterized by an increased inflammatory ­burden, which plays a significant role in its ­pathophysiology^5^. Inflammatory processes contribute to the damage and repair mechanisms in stroke, highlighting the importance of ­considering inflammation in stroke prognosis. Besides stroke, other ­neurological diseases are also associated with varying degrees of inflammation. For example, the serum C-reactive protein to albumin ratio has been identified as a reliable marker of ­inflammation in conditions such as diabetic neuropathy^6^.

The prognostic nutritional index (PNI) is a measure that reflects patients’ nutritional status and overall health ­condition^7,8^. PNI is calculated by combining serum albumin levels and total lymphocyte count, and it has been shown to have ­prognostic value, particularly in chronic diseases and malignancies^9-11^. Nutritional indexes are known to be ­associated with ­inflammatory conditions. The PNI, which combines serum albumin levels and total lymphocyte count, has been reported to correlate with inflammatory ­conditions such as ­diabetic nephropathy and infections^12,13^. This ­association ­underlines the potential utility of PNI in studying ­inflammatory ­processes in stroke.

This study aimed to evaluate the impact of PNI on ­mortality in patients with ischemic stroke and to investigate its potential use as a tool for predicting patient prognosis.

## METHODS

This retrospective, observational study was conducted on patients diagnosed with acute ischemic stroke in the emergency ­department of Kartal Dr. Lütfi Kırdar City Hospital between January 1, 2022, and January 1, 2023. Ethical approval for the study was obtained from the Kartal Dr. Lütfi Kırdar City Hospital Ethics Committee on August 9, 2023, with ­decision number 2023/514/255/5. During the specified period, patients who were 18 years and older, diagnosed with acute ischemic stroke, and had the necessary laboratory and clinical data in their hospital records were included in the study. Patients with active ­inflammatory disease, known malignancy or ­hematological ­disease, severe hepatic and renal dysfunction, patients under 18 years of age, patients diagnosed with hemorrhagic stroke, and those with incomplete or insufficient medical records were excluded.

The PNI is calculated by combining serum albumin levels and the total lymphocyte count. The PNI is calculated using the following Formula 1^14^:


(1) $$\begin{array}{l} 10\times \text{serum albumin}(\text{g/dL})+0.005\times \text{total lymphocyte count}\\ \left({\text{per mm}}^{3}\right) \end{array}$$

The demographic data, laboratory results, hospital records, and clinical data of the patients were obtained retrospectively from electronic health records. Serum albumin levels and total lymphocyte counts were evaluated using the initial ­laboratory results at hospital admission. The primary outcome of the study was in-hospital mortality. The mortality status of the patients was confirmed through hospital records and electronic health systems.

### Statistics

Statistical analyses were conducted using the SPSS software for Windows (Version 29, Chicago, IL, USA) and MedCalc (Version 20.104, MedCalc Software Ltd., Ostend, Belgium). Descriptive statistics summarized the data, with counts and ­percentages reported for categorical variables and mean±­standard deviation or median (interquartile range [IQR] 25th–75th) values for continuous variables. The Kolmogorov-Smirnov test and ­histograms assessed the normality of the data distribution. Group comparisons were made using the Pearson chi-square test for categorical variables (or Fisher's exact test when assumptions were violated) and the Student's t-test or Mann-Whitney U test for continuous variables. All analyses were two-sided, with a significance level of <0.05. Receiver operating characteristic curves were drawn for diagnostic validity tests, and sensitivity, specificity, positive likelihood ratios, and negative likelihood ratios were calculated for the cut-off values with the highest Youden index for the parameters with statistical significance.

## RESULTS

The study included 176 patients, divided into survivor (93.2%, n=164) and deceased (6.8%, n=12) groups based on ­mortality outcomes. Demographic characteristics, vital parameters at admission, comorbidities, and stroke interventions for these patients are detailed in Table 1. The median age of the cohort was 71 years (IQR 61–85), with no statistically significant age difference between the groups (p=0.713). The cohort consisted of 55.1% males, with no significant difference in gender distribution between the groups (p=0.712).

**Table 1:** Comparison of demographic characteristics, vital parameters, comorbidities, and acute stroke treatments between survivor and deceased groups.

Characteristic	Total (n=176)	Survivor (n=164)	Deceased (n=12)	p
Age (IQR)	71 (61–85)	71 (61–78)	81 (68.5–86.25)	0.713
Male gender, n (%)	97 (55.1)	91 (55.5)	6 (50)	0.712
Systolic BP (mmHg)	157.9±32.1	157.6±32.1	162.8±31.7	0.591
Diastolic BP (mmHg)	84±17	83.9±16.9	84.8±19.2	0.860
Pulse rate (/min)	85.6±15.8	85.3±17.8	89.4±27.2	0.458
Temperature (C°)	36.5 (36.3–36.7)	36.5 (36.3–36.7)	36.6 (36.4–36.8)	0.164
AF, n (%)	37 (21)	31 (18.9)	6 (50)	0.011
Hypertension, n (%)	127 (72.2)	117 (71.3)	10 (83.3)	0.371
DM, n (%)	70 (39.8)	65 (39.6)	5 (41.7)	0.890
HL, n (%)	13 (7.4)	13 (7.9)	0 (0)	0.311
CAD, n (%)	40 (22.7)	35 (21.3)	5 (41.7)	0.105
Thrombolytic therapy, n (%)	14 (8)	12 (7.3)	2 (16.7)	0.248
Mechanical thrombectomy, n (%)	32 (18.2)	30 (18.3)	2 (16.7)	0.888

AF: atrial fibrillation; CAD: coronary artery disease; DM: diabetes mellitus; HL: hyperlipidemia; IQR: interquartile range.

The mean systolic and diastolic blood pressures were ­similar between the survivor (157.6±32.1 and 83.9±16.9 mmHg, respectively) and deceased (162.8±31.7 and 89.4±27.2 mmHg, respectively) groups (p=0.591 and p=0.860, respectively). The average heart rate among all patients was 85.6±15.8 bpm, and there was no significant difference between the groups (p=0.458). Additionally, no significant difference in body temperature was observed between the groups (p=0.164).

Atrial fibrillation was significantly more common in the deceased group (50%, n=6) compared to the survivor group (18.9%, n=31) (p=0.011). However, there were no ­statistically significant ­differences in the prevalence of hypertension, ­diabetes, ­hyperlipidemia, and coronary artery disease between the groups (p=0.371, p=0.890, p=0.311, and p=0.105, ­respectively). Furthermore, the frequencies of intravenous thrombolytic ­therapy and mechanical ­thrombectomy application did not differ ­significantly between the groups (p=0.248 and p=0.888, respectively).

Table 2 presents the laboratory data and PNI values at admission. No statistically significant differences were observed in median glucose, creatinine, and blood urea nitrogen (BUN) levels between the groups (p=0.470, p=0.276, and p=0.250, respectively). The median lymphocyte count was significantly higher in the survivor group (1,353 [IQR 984–1,968]/mm³) compared to the deceased group (660 [IQR 462–1,188]/mm³) (p=0.009). The median albumin level was significantly lower in the deceased group (3.31 [IQR 2.67–3.4] g/dL) compared to the survivor group (3.74 [IQR 3.39–4.21] g/dL) (p<0.001). The median PNI was significantly higher in the survivor group (46.05 [IQR 39.1–51.3]) compared to the deceased group (36.7 [IQR 28.7–40.5]) (p<0.001).

**Table 2:** Comparison of laboratory data and prognostic nutritional index between survivor and deceased groups.

Parameter	Survivor (n=164)	Deceased (n=12)	p
Glucose (mg/dL, IQR)	137 (107.3–175.8)	138.5 (116–196.5)	0.470
Creatinine (mg/dL, IQR)	0.91 (0.77–1.1)	0.91 (0.79–1.1)	0.276
BUN (mg/dL, IQR)	17 (14–23.8)	20.5 (14.8–23.8)	0.250
Lymphocyte count (/mm3, IQR)	1,353 (984–1,968)	660 (462–1,188)	0.009
Albumin (g/dL, IQR)	3.74 (3.39–4.21)	3.31 (2.67–3.4)	<0.001
PNI (IQR)	46.05 (39.1–51.3)	36.7 (28.7–40.5)	<0.001

PNI: prognostic nutrition index; IQR: interquartile range.

The diagnostic validity analysis of PNI for predicting ­mortality in acute stroke patients yielded an area under the receiver ­operating characteristic (AUROC) of 0.791 (95% confidence interval [CI] 0.723–0.848) (p=0.0002), as shown in Table 3. The cut-off value with the highest Youden index was determined to be ≤41.92. At this cut-off, the sensitivity was 91.67% (95%CI 61.5–99.8), the specificity was 65.85% (95%CI 61.9–76.5), the positive likelihood ratio was 3.01 (95%CI 2.26–4.01), and the negative likelihood ratio was 0.12 (95%CI 0.018–0.79).

**Table 3:** Prognostic nutritional index prediction of mortality outcomes.

AUROC (95%CI)	p	Cut-off	Sensitivity (95%CI)	Specificity (95%CI)	+LR (95%CI)	-LR (95%CI)
0.791 (0.723–0.848)	0.0002	≤41.92	91.67 (61.5–99.8)	65.85 (61.9–76.5)	3.01 (2.26–4.01)	0.12 (0.018–0.79)

CI: confidence interval; +LR: positive likelihood ratio; -LR: negative likelihood ratio.

## DISCUSSION

The significant findings of this study indicate that low PNI values are associated with a high risk of in-hospital mortality in patients with acute ischemic stroke. This suggests that PNI could be a valuable prognostic tool in stroke management.

The PNI is a measure that combines serum albumin ­levels and the total lymphocyte count. Serum albumin is ­considered an indicator of protein reserves in the body, with low levels generally associated with poor nutritional status and chronic illnesses^15,16^. Total lymphocyte count reflects the functioning of the immune system, and low levels may indicate ­immunodeficiency^17,18^. Together, these two components are used to assess the ­overall nutritional and inflammatory status of patients. Good ­nutritional status can accelerate recovery, reduce the risk of infections, and improve the overall prognosis of stroke patients. Conversely, malnutrition can negatively impact these processes, increasing the risk of complications and delaying recovery^19,20^.

In this study, low PNI values were found to be associated with mortality. Similarly, in the study by Xiang et al., low PNI values were reported to be associated with poor clinical ­outcomes at 3 months in patients with acute ischemic stroke. They ­highlighted that PNI is an independent prognostic ­indicator in patients receiving intravenous thrombolysis, and low PNI values are associated with poor prognosis^21^. In a study conducted in China, low preoperative PNI values were shown to increase the risk of perioperative ischemic stroke in patients undergoing ­non-cardiac surgery. Patients with PNI <38.8 had a higher incidence of perioperative ischemic stroke ­compared to those with PNI ≥38.8^22^. Nergiz et al. demonstrated that low PNI values were associated with in-hospital ­mortality, ­prolonged hospital stays, and a higher risk of infection in patients with acute ischemic stroke^23^. Wang et al. showed that, in young stroke patients, low PNI values were associated with high ­levels of inflammation and were an independent risk factor for a poor 90-day prognosis^24^.

Our findings suggest that incorporating PNI into routine clinical practice could significantly impact the management of patients with acute ischemic stroke. By identifying patients with low PNI values, clinicians can use PNI as a simple, cost-effective tool to stratify stroke patients based on their nutritional and inflammatory status. This early identification of high-risk patients allows for more targeted interventions. Additionally, given the association between low PNI and poor outcomes, early ­nutritional intervention strategies can be implemented to improve the overall prognosis. Understanding the ­nutritional and inflammatory status of stroke patients can help tailor ­treatment plans that address these specific issues, potentially leading to better outcomes. Moreover, patients identified as high-risk through PNI can be monitored more closely during their hospital stay and in follow-up visits, ensuring timely ­interventions if their condition deteriorates.

This study has several limitations. First, the retrospective nature of the study may introduce biases and inaccuracies in data collection and recording. Second, the study was conducted at a single center, limiting the generalizability of the results. Third, only in-hospital mortality was assessed; long-term ­outcomes and other complications were not evaluated. Finally, to definitively establish the role of PNI in predicting stroke prognosis, larger, multicenter, and prospective studies are needed.

## CONCLUSION

This study demonstrates that there is a significant association between low PNI values and increased in-hospital mortality in patients with acute ischemic stroke. While PNI shows promise as a prognostic indicator, it cannot be definitively assigned as a predictor of mortality due to the retrospective design of the study. Nevertheless, incorporating PNI into clinical practice may aid in the early identification of high-risk patients and the optimization of treatment strategies. Further prospective studies are needed to validate these findings and explore the broader clinical applications of PNI.

## ETHICAL APPROVAL

This study was approved by the local ethics committee (ethics committee ruling number: 2023/514/255/5, date: 09.08.2023).
